# Urgent Carotid Surgery: Is It Still out of Debate?

**DOI:** 10.1155/2012/536392

**Published:** 2012-03-19

**Authors:** C. Battocchio, C. Fantozzi, L. Rizzo, F. Persiani, S. Raffa, M. Taurino

**Affiliations:** Azienda Ospedaliera Sant'Andrea, Facoltà di Medicina e Psicologia, Sapienza-Università di Roma, 00189 Roma, Italy

## Abstract

Patients with symptomatic tight carotid stenosis have an increased short-time risk of stroke and an increased long-term risk of ischaemic vascular events compared with the general population. The aim of this study is to assess the safety, efficacy, and limitations of urgent CEA or CAS, in patients with carotid stenosis greater than 70% and clinically characterized by recurrent TIA or brain damage following a stroke (<2.5 cm). This study involved 28 patients divided into two groups. Group A consisted of sixteen patients who had undergone CEA, and group B consisted of twelve patients who had undergone CAS. Primary endpoints were mortality, neurological morbidity (by NIHSS) and postoperative hemorrhagic cerebral conversion, at 30 days. Ten patients (62.5%) of group A experienced an improvement in their initial neurological deficit while in 4 cases (26%) the deficit remained stable. Two cases of neurologic mortality are presented. At 1 month, 9 patients (75%) of group B experienced an improvement in their initial neurological deficit while 3 patients (25%) had a neurological impairment. Urgent or deferred surgical or endovascular treatment have a satisfactory outcome considering the profile in very high-risk patient population. Otherwise in selected patients CEA seems to be preferred to CAS.

## 1. Introduction

Ipsilateral >50% carotid stenosis is found in about 10% of carotid territory ischaemic strokes and in about 15% of TIAs (transitory ischemic attack), and is associated with a particularly high risk of recurrent stroke [1–3], both in the acute phase and long term [4]. Recent studies showed that 4−20% of TIA patients will have a stroke within 90 days after a TIA, half within the first 2 days [5, 6]. Reanalysis of controlled trials of CEA (carotid endarterectomy) indicated that surgery conferred the greatest benefit when performed in the first 2 weeks following the index symptoms, perhaps as early as 48 h after the index event [7, 8]. Delaying intervention quite probably means that patients are better selected and this could guarantee better early outcomes, but this delay can also result in an interval stroke rate of 9–15% [9]. In the past few years CAS (carotid artery stenting) has emerged as a treatment alternative to CEA. A recent study found that early CAS might be associated with an increased risk for stroke and death [10]. In fact CAS, in the acute stage, remains challenging because of the limited therapeutic window and risk of hyperperfusion syndrome or cerebral hemorrhagic infarction after revascularization [11]. The real goal of early treatment is to stop the plaque embolization from a vulnerable lesion at the carotid bifurcation [12]. Another important question is: is there a patient at higher risk of neurological impairment in the short term? The ABCD^2^ score is a prognostic score that we, retrospectively, used to validate our practice [13]. This retrospective study investigates the safety, efficacy, and limitations of urgent CEA or CAS for symptomatic patients with recent first or recurrent TIA or minor stroke, bearing severe carotid artery stenosis.

## 2. Methods

This was a retrospective review of two groups of patients: group A, with patients treated with open surgery, and group B, with patients treated with endovascular technique. In our institution the guidelines of treatment do not consider BMT, so a control group is not available. All patients were observed in emergency department (ED) by a neurologist and a vascular surgeon. Cerebral CT scan or MRI was performed in all patients before and after the treatment. If CT scan or MRI ruled out hemorrhagic events, intravenous heparin therapy was administered immediately. Demographic data, including age, sex, symptom details, risk factors, medications, and operative data were collected for all patients. The National Institutes of Health Stroke Scale (NIHSS) was used for neurological assessment; the score was recorded routinely before, 24 h, and 3 days after treatment. Ecocolor Doppler (ECD) was performed in all patients to evaluate the carotid bifurcation (type of plaque and haemodynamics of the lesion). Anatomy of aortic arch and supra-aortic trunks were also evaluated with angio-CT o MRI in all cases. All patients underwent transcranial Doppler (TCD) before surgery to evaluate the presence and frequency of microembolic signal (MES), to detect the middle cerebral artery (MCA) patency, and to test the intraoperative clamping tolerance.

The emergency treatment was performed in patients with very tight stenosis and clinically characterized by recurrent TIA or stroke with little cerebral damage (<2.5 cm). For patients with different clinical picture, we had the possibility to keep time to prepare them in the best way for surgery.

Only one patient was undergoing preoperative fibrinolytic therapy and then she died of haemorrhagic postoperative cerebral infarction. All patients received intravenous heparin before carotid intervention.

Patients were excluded if cerebral ischemic lesions were greater than 2.5 cm, if there was loss of consciousness, if there were signs of intracranial hemorrhage, and if the patients or family did not give informed consent.

**Group A**

Sixteen patients who had undergone CEA after TIA, minor or major stroke, from October 2004 to August 2010, were identified. Clinical characteristics of this group of patients are shown in Table 1. In 13 patients a patch angioplasty, in 2 cases a direct suture of arteriotomy, and in 1 case an eversion CEA were performed.

A Pruitt Inahara shunt has been used in 8 patients. For interventions performed under general anaesthesia, it has been placed when, during clamping, the DTC signal showed a reduction of 2/3 of middle cerebral artery flow, while for interventions performed under local anaesthesia, it has been used when, during clamping, a clinical deficit appeared. In suitable patients CEA was undertaken on the next available operating room, with a preference for local anaesthesia.

**Group B**

Twelve patients who had undergone CAS after TIA minor or major stroke, from September 2004 to February 2011, were identified. Clinical characteristics of this group of patients are shown in Table 1. The cerebral protection was performed in all cases with distal embolic protection device. In 8 cases the CAS was performed with femoral access, in 4 patients a cervical cutdown was used. In suitable patients CAS was undertaken on the next available operating room by vascular surgeon, in local anaesthesia.

CEA contraindications were high-risk patients, hostile neck, and high bifurcation. CAS contraindications were difficult access anatomy, very tight stenosis, and soft or very calcified lesions (Table 2) [14].

In absence of contraindications for CAS or CEA the operative technique was chosen according to surgeon's preference.

All patient were observed in critical care unit after operation.

Retrospectively we applied ABCD^2^ score [13] to validate or not our therapeutic strategy (the appendix).

Patients were followed up for 30 days after their clinical presentation. Stroke, TIAs, deaths, and hospitalization for cardiovascular events were identified for all patients. The primary outcome was recurrent TIA or minor stroke or stroke occurring within 30 days of TIA or minor stroke presentation. Secondary outcome were cardiovascular events, requiring hospitalization, and death.

## 3. Statistical Analysis

Patients were divided for analysis into subgroups according to perioperative characteristics and were compared with respect to the occurrence of postoperative neurologic events or NIHSS score variation (≥1).

Statistical analysis was performed with chi-square test and Mann-Whitney test.

## 4. Results

From February 2003 to February 2011 we treated “early” 28 patients with carotid disease, for a total of 16 TEA (57%) and 12 CAS (43%). A summary of the clinical results for all patients group are given in Table 1. The mean age was 70 years (min 60, max 80) in group A and 75 years (min 70, max 80) in group B. The interval from symptom onset to arrival at the emergency department did not differ between groups. The prevalence of common vascular risk factors was also similar between two groups. All patients had mild to moderate neurologic deficit: median NIHSS score of 7.4 in group A and 4.4 in group B (Table 3). This is the only significant difference between the two groups. At admission, early signs of cerebral ischemia were present at CT or MR imaging in 10 (62.5%) of 16 patients in group A and in 7 (58%) of 12 patients of group B. The mean grade of stenosis was 77.5% (from 70 to 85) for group A and 75% (from 70 to 80) for group B. Type of plaque at ECD was I in 5 cases, II in 5 cases, III in 3 cases, IV in 1 cases, and V in 2 cases in group A. Type of plaque at ECD was I in 1 case, II in 3 cases, III in 7 cases, IV in 1 cases, and V in 0 cases in group B. TCD showed relevant MES in 4 patients of group A and in all patients of the group B. TCD depicted 16 patients with possible clamp ischemia (8 in group A and 9 in group B). The CT or MR imaging revealed 4 cases of contraindication to endovascular femoral procedure (Leriche, tortuosity, and hostile arch).

**Group A**

Successful CEA was achieved in all cases (100%). No intraprocedural neurologic complication occurred in all but two. Procedural success, defined as absence of new cerebrovascular events at discharge (including major stroke, minor stroke, or TIA) was assessed at 87.45%. One female and one male patient with crescendo TIA experienced an in-hospital new neurologic event: stroke due to hemorrhagic transformation of ischemic lesion. A Pruitt Inahara shunt has been used in one patient. These patients died one month after coma. Other nonneurological adverse event was one acute myocardial infarction (AMI). An haematoma drainage in the postoperative period was never necessary. The postoperative NIHSS score at hospital discharge showed that in 10/16 patients (62.5%) the neurological deficit improved (decrease of NIHSS score >1), in 4/16 patients (25%) the NIHSS score remained unchanged (decrease of NIHSS score <1), and in 2 cases (12.5%) the neurological deficit showed an impairment (coma). At 30 days NIHSS score showed that in 11/16 patients (68.7%) the neurological deficit improved (decrease of NIHSS score >1), in 4/16 patients (25%) the NIHSS score remained unchanged (decrease of NIHSS score <1), and in 2 cases (12.5%) the neurological deficit showed an impairment (coma) (Tables 4 and 5).

No new ischemic lesions were detected by CT or MR postoperative imaging although the NIHSS preoperative score was significantly higher than in group B.

**Group B**

Successful CAS was achieved in all cases (100%). In 8 cases a Boston Scientific Carotid Wallstent, in 3 cases an Invatec Cristallo Ideale stent, and in 1 case an EV3 Protégé RX carotid stent were implanted.

No intraprocedural neurologic complication occurred, while the minor complications that occurred during the procedure were ICA vasospasm in 2/12 cases (16%) and severe bradicardia and hypotension in 1/12 case (8%). Procedural success, defined as absence of new cerebrovascular events at discharge (including major stroke, minor stroke, or TIA) was assessed at 76%. Three patients experienced an in-hospital new neurologic event (2 minor stroke 16%; 1 stroke 8%). Other nonneurological adverse events were a persistent bradicardia and an AMI. The postoperative NIHSS score at hospital discharge showed that in 8/12 patients (66%) the neurological deficit improved (decrease of NIHSS score >1), in 1/12 patients (8%) the NIHSS score remained unchanged (decrease of NIHSS score <1) and in 3/12 cases (24%) the neurological deficit showed an impairment (2 minor strokes 16%; 1 stroke 8%), in two cases without new ischemic cerebral lesions. In 3 patients new ischemic lesions were detected by CT or MR postoperative imaging, 2 of these were asymptomatic although the NIHSS preoperative score was significantly lower than in group A.

At 90 days NIHSS score showed that in 9/12 patients (75%) the neurological deficit improved (decrease of NIHSS score >1) and in 3/12 cases (24%) the neurological deficit showed an impairment (2 minor strokes 16%; 1 stroke 8%) (Tables 4 and 5).

## 5. Discussion

This study demonstrates the safety and the efficacy of early CEA or CAS after TIA (within 24–48 hours) or stroke (within 14–28 days).

Effective and early management of patients with acute symptoms due to carotid stenosis is still the subject of debate. The inability to predict who is at higher early risk of a recurrent stroke after a cerebrovascular event (TIA or stroke) may explain the variation in management of acute stroke comparing physician to physician and institution to institution [12].

Improving outcomes of carotid surgery have recently focused on the appropriate timing of carotid intervention in the setting of new-onset neurological deficit. The increased risks of reperfusion injury and conversion to hemorrhagic infarction have led to the historical recommendation of delayed CEA in those presenting with acute stroke [15–17]. However, several high-volume centers have documented the safety and efficacy of early intervention in preventing recurrent strokes in carefully selected patients [18, 19]. More specifically, patients presenting with fluctuating and potentially reversible neurological deficits may benefit from the removal of an embolic source and/or salvage the at-risk areas of the brain [18, 20]. Comparing medical therapy to surgical intervention in those presenting with nondisabling stroke and TIA, nonoperative therapy carried a 54% rate of combined death and worsening neurological condition whereas the combined death and stroke rate in the surgical group was 7% [21]. The reported stroke risks highlight how early removal of the carotid plaque, that is considered to be the embolic source, could be crucial in stroke prevention [12]. Rothwell et al. [22] have recently analyzed pooled data from the European Carotid Surgery Trial and North American Symptomatic Carotid Endarterectomy Trial (a total of 5893 patients). The analysis of long-term stroke prevention (per 1000 CEAs at 5 years), in relation to the delay in surgery, clearly showed that benefit from surgery decreased rapidly with time elapsed since the last neurological symptoms. Profit from endarterectomy seems to depend not only on the degree of carotid stenosis, but also on delay in surgery. Recent data have conclusively shown that patients benefit when intervention is performed within 2 weeks from the index event in order to prevent recurrent stroke, even if the procedural risk may be higher [4, 22]. The SPREAD guidelines also clearly state that carotid surgery is recommended as early as possible—within 2 weeks of the event—for patients with TIA, minor stroke, or stabilized neurological deficit with normal CT scanning or minimal lesions (Grade A recommendation) [23]. In a prospective study of patients presenting with acute symptomatic high-grade internal carotid artery stenosis, the Oxford Vascular Study Group recently observed that for patients with TIA the 7-day, 30-day, and 3-month risks of stroke were 8%, 12%, and 17% respectively, while for patients presenting with minor stroke (NIH stroke score <3) parallel data were 12%, 15%, and 19% [24]. These data suggest that acute stroke or TIA should be considered as medical emergencies that require rapid evaluation and rapid targeting of treatment.

The decision to proceed with an urgent intervention for those presenting with acute neurological deficit is challenging and requires very careful attention. Rothwell et al. in 2007 have reported a prospective study that shows how an early treatment (medical and/or surgical) of all patients presenting with TIA or minor stroke can prevent about 80% of early recurrent stroke [25]. Although the timing of intervention may be debatable, the mode of intervention is even more controversial.

We demonstrated the safety of CEA in this series of high-risk/complex carotid cases enrolled and treated soon after the onset of symptoms, as shown by the little percentage of neurological complications during the procedure, a high procedural success rate, and the very low rate of minor cerebrovascular events in the postoperative period. The clinical outcome at 1 month of a 12.5% stroke/mortality rate and 12.5% rate for all neurological events is satisfactory considering the very high-risk profile of the patient population.

An Italian multicenter study, Surgical Treatment of Acute Cerebral Ischemia (STACI) [26], has recently shown that patients, whose neuroimaging studies document a recent, limited cerebral infarction in the early hours after a stroke, can safely undergo very early CEA (1.5 days after the stroke). This study underlines that if patients are strictly selected for early CEA after an acute stroke, early surgery gains similar results to elective surgery. Capoccia et al. in a recent study suggest that minimizing the time for intervention not only reduces the risk of recurrence but can also improve neurologic outcome [27]. Gertler et al. [18] reviewed the results of CEA in neurologically unstable patients with significant carotid stenosis and presenting with crescendo TIA continuing despite heparin or stroke in evolution. The condition of all but one (2.7%) was either improved or stabilized after operation. They recommended early intervention of neurologically unstable patients to improve the outcome in those patients. Huber et al. [28] demonstrated, through pre- and postoperative diffusion-weighted (DW) and perfusion-weighted (PW) MR, a reduction of the cerebral ischemic lesion in the patients operated on. Speculatively, these data might suggest a role of surgical reperfusion in rapidly improving the clinical course even 24 to 48 hours after stroke onset. Obviously, this speculation would need to be tested in large number of patients studied with DW and PW MR.

In the absence of scientific proof from conclusive randomized trials, and despite the negative results of Endarterectomy versus Angioplasty in Patients with Symptomatic Severe Carotid Stenosis (EVA-3S) [29] and Stent-Protected Angioplasty versus Carotid Endarterectomy (SPACE) [30, 31] favoring CEA over CAS in symptomatic severe carotid artery stenosis, many interventionalists consider both modalities to be equivalent [21]. With growing experience in endovascular treatment, CAS has been proposed as an alternative to CEA, but data regarding the outcome of patients with acute stroke undergoing urgent endovascular surgery are still scarce. The main concern about CAS in urgent cases is that while with CEA the plaque is completely removed, after stenting it is only remodelled and its stabilization is essential to avoid later embolic events [12].

We demonstrated the safety of CAS in this series of high-risk/complex carotid cases enrolled and treated soon after the onset of symptoms, as shown by the little percentage of neurological complications during the procedure, a high procedural success rate, and the very low rate of minor cerebrovascular events in the postoperative period. The clinical outcome at 1 month of a 0% stroke/mortality rate and 25% rate for all neurological events are satisfactory considering the very high-risk profile of the patient population. In this study an open-cell stent was used in one out of 12 cases.

Setacci et al. [12] enrolled 57 patients in a prospective registry studying the role of protected CAS in those presenting with symptomatic carotid lesion. Twenty-four patients (42%) had TIA and were intervened upon within 24 to 48 hours from the last episode, while the remaining 33 (58%) patients who presented with stroke and intervention were delayed to 14 to 30 days. At 30 days, 1 patient died (1.7%) and 2 suffered postoperative TIAs (3.5%). They concluded that CAS with embolic protection is a feasible and safe alternative to CEA in the acute setting.

An important consideration during CAS is the concern of scaffolding of vulnerable plaques and the risk of distal embolization. The role of stent designs as well the use embolic protection device will remain to be determined by the outcome of ongoing and yet-to-start randomized trials [21]. We wait for the results of Submarine II Registry to evaluate the safety of CAS in symptomatic stroke patients using either a closed- or an open-cell stent [12]. At present, sophisticated imaging techniques such as pixel density analysis and elastography at duplex examination [32, 33], magnetic resonance imaging for tissue characterization [34, 35], or local temperature probes [36, 37] all hold promise for the noninvasive identification of vulnerable plaques and the detection of silent atheroma.

Mussa et al. [21] obtained a confirmatory study in all patients undergoing CEA, and in the last few years, they have relied on images from MRA and CTA to guide their indications and more remarkably, the mode of intervention. A potential advantage of MRA and CTA over conventional angiography would be their ability to image the residual lumen and may aid the characterization of “vulnerable” plaque to evaluate stroke risk. Furthermore, with the increasing role of carotid angioplasty and stenting, these imaging modalities provide a detailed map for the aortic arch and the cervical course of the internal carotid artery.

In our study of the 28 patients, 20 (71%) showed either total or partial resolution of symptoms. One patient with TIA deteriorated after intervention. Our combined death and stroke rate of 17.8% is comparable with that of others.

In conclusion, our study demonstrated that early treatment with CEA or protected carotid stenting is both feasible and safe in selected patients with first episode or recurrent TIA or minor stroke. This preliminary study in a limited series of patients revealed that an urgent endovascular approach has a satisfactory outcome considering the very high-risk profile of the patient population, but CEA remains the gold standard.

Given that the ABCD^2^ score seems to constitute a valuable tool able to identify in the ED the subgroup of TIA or minor stroke patients which is at greatest need for emergent evaluation and effective treatment, the applicability of the former score in clinical practice may raise certain potential therapeutic implications.

## Figures and Tables

**Table 1:** Comparison of clinical characteristics and results of clinical workup between patients in surgical group and those in endovascular group.

Parameter	Surgical group (*n* = 16)	Endovascular group (*n* = 12)
Sex		
Female	5	3
Male	11	9
Age (y)		
Mean	70	75
Vascular risk factors		
Hypertension	7 (43)	5 (41)
Current cigarette smoking	8 (50)	7 (58)
Diabetes mellitus	6 (37)	8 (66)
Hypercholesterolemia	9 (56)	6 (50)
Heart disease	3 (18)	5 (41)
History of amaurosis fugax, retinal infarct, transient ischemic attack, or stroke	4 (25)	3 (25)
Neurological status during treatment		
Asymptomatic (TIA)	7 (44)	3 (25)
Symptomatic stable (minor stroke)	3 (18)	5 (41)
Symptomatic stable (major stroke)	6 (37)	4 (33)
Median NIHSS score	7.4	4.4
Time from onset of symptoms to treatment		
Emergency (<24 h)	13 (81)	11 (91)
Urgency (<7 days)	3 (18)	1 (9)
Early neurological results		
Improvements (>1 NIHSS)	10 (63)	9 (75)
Stable	4 (25)	—
Impairment (>1 NIHSS)	2 (12.5)	3 (25)
Early death	2 (12.5)	—
New ischemic lesions p.o.	—	3 (25)
Cerebral hemorrhage	2 (12.5)	—

Note: numbers in parentheses are percentages.

**Table 2:** Nicolaides plaque classification.

Plaque	Morphologic characterization
Type 1	Uniformly echolucent plaque
Type 2	Predominantly echolucent plaques with less than 50% echogenic areas
Type 3	Predominantly echogenic plaques with less than 50% echolucent areas
Type 4	Uniformly echogenic plaques
Type 5	Could not be classified (heavy calcification and acoustic shadows)

**Table 3:** 

	NIHSS		
	surgical	endovascular	*P* value*
At admission	7.4	4.4	<0.0001

*Chi-square test for trend.

**Table 4:** 

Parameter	Surgical group	Endovascular group	*P* value*
Number of patients	16	12	

Early neurological results			
Improvement (>1 NIHSS)	10 (63.5%)	9 (75%)	0.15
Stable	4 (25%)	0	
Impairment (<1 NIHSS)	2 (12.5%)	3 (25%)	

*Chi-square test for trend.

**Table 5:** 

	NIHSS		
	At admission	Postoperative	*P* value*
Surgical group	7.4	5.5	0.21
Endovascular group	4.4	3.4	0.36

*Mann-Whitney test.

## Appendix

### ABCD^2^ Score

For the needs of the present study, an investigator (C. Fantozzi) blinded to the follow-up events retrospectively reviewed both the ED and hospital records of all 28 patients. The 7-point ABCD^2^ score (age (<60 years = 0, ≥60 years = 1); BP (systolic ≤140 mm Hg and diastolic ≤90 mm Hg = 0, systolic >140 mm Hg and/or diastolic >90 mm Hg = 1); clinical features (unilateral weakness = 2, speech disturbance without weakness = 1, other symptom = 0); duration of symptoms (<10 minutes = 0, 10 to 59 minutes = 1, ≥60 minutes = 2) and diabetes [1] was computed in 20 cases. Clinical features were categorized as motor weakness (focal, usually unilateral, weakness of one or more of face, arm, hand, or leg) versus speech disturbance (defined as dysarthria or dysphasia or both) versus all other symptoms (numbness, change in vision, dizziness or vertigo, and gait disturbance) according to the OCSP definition of the ABCD^2^ score. Seven patients with unavailable BP recordings (*n* = 2) or duration of TIA symptoms (*n* = 5) at the ED records were excluded from further evaluation.
